# Incidence, Management, Prevention and Outcome of Post-Operative Atrial Fibrillation in Thoracic Surgical Oncology

**DOI:** 10.3390/jcm9010037

**Published:** 2019-12-23

**Authors:** Iacopo Fabiani, Alessandro Colombo, Giulia Bacchiani, Carlo Maria Cipolla, Daniela Maria Cardinale

**Affiliations:** 1 Cardioncology Unit, European Institute of Oncology, I.R.C.C.S, 20141 Milan, Italy; iacopo.fabiani@ieo.it (I.F.); alessandro.colombo@ieo.it (A.C.); giulia.bacchiani@ieo.it (G.B.); 2 Cardiology Division, European Institute of Oncology, I.R.C.C.S., 20141 Milan, Italy; carlo.cipolla@ieo.it

**Keywords:** atrial fibrillation, brain natriuretic peptide, N-terminal-pro-natriuretic peptide, thoracic surgery, cancer, thrombosis, cardiotoxicity

## Abstract

Atrial fibrillation (AF) is a common supraventricular arrhythmia, a recognized risk factor for ischemic stroke, as a potential driver for heart failure (HF). Cancer patients have an increased risk for AF, even not including any cancer-specific treatment, as surgery or chemotherapy. The mechanism is multifactorial, with inflammation and changes in autonomic tone as critical actors. Commonly, AF is a recurrent complication of the post-operative period in cancer surgery (especially thoracic). Recent papers confirmed a significant incidence of post-operative (non-cardiac surgery) AF (PAF), partially mitigated by the use of prophylactic (rate o rhythm control) treatments. A relevant difference, in terms of mean hospitalization time, emerges between patients developing PAF and those who do not, while long term impact remains a matter of debate, due to several potential confounding factors. Besides clinical predictors, structural (i.e., echocardiographic) and bio-humoral findings may help in risk prediction tasks. In this respect, pre-operative natriuretic peptides (NPs) concentrations are nowadays recognized as significant independent predictors of perioperative cardiovascular complications (including PAF), while elevated post-operative levels may further enhance risk stratification. The aim of the present paper is to trace the state of the art in terms of incidence, management, prevention, and outcome of PAF in the field of thoracic surgical oncology.

## 1. Introduction

Atrial fibrillation (AF) is a common sustained arrhythmia (1.5–2% of the general population) [1], with a relevant weight in terms of morbidity and mortality.

Besides patients with known heart disease, a high rate of AF is reported in the oncologic setting [2]. 

As reported in the literature, there is a high rate of AF incidence among patients with cancer, a condition which poses relevant prognostic and therapeutic implications [3,4,5,6,7]. Even with considering the improvement in cancer therapy and survival rates, this associated comorbidity represents a relevant public health condition, due to cancer epidemiology and an aging population.

So far, a global but also tailored cardio-oncological evaluation of the problem is expected [8,9].

Post-operative atrial fibrillation (PAF) is a common finding in the field of surgical oncology, and the most common and historical association between these two pathophysiological entities. 

The prevalence of PAF results in 16–46% after cardiothoracic surgery and 0.4–12% after non-cardiothoracic surgery [10]. 

In this specific setting, especially in thoracic surgery, knowledge of PAF is mainly based on small retrospective studies, and high-quality prospective studies are still lacking.

PAF is associated with longer hospital stays and readmissions, as well as a risk of stroke [11,12,13].

The aim of the present review is to trace the state of the art in terms of incidence, prediction, prevention, and outcome of PAF in the field of surgical thoracic oncology.

Specific indications on acute and chronic treatments, as well as the task of anti-coagulation, fall behind the scope of the present review.

## 2. Definitions

By electro-physiologic definition, an ECG recording that demonstrates AF, at least for thirty seconds, or for the duration of the ECG recording (Class I—level of evidence LOE C) identifies PAF, while, according to a clinical definition, PAF is defined by a significant AF episode in the operative setting requiring drug treatment and/or influencing the hospitalization (Class I—LOE C) [14]. 

Both electrophysiological and clinical criteria should be considered.

### 2.1. PAF in Thoracic Surgery Setting: Epidemiology 

AF is a common supra-ventricular arrhythmia after pulmonary (and esophageal) surgery. Early papers (early to mid-1990s) were the first to report increased rates of AF after thoracic surgery [15]. 

However, the incidence of PAF varies widely, according to surgical conditions (with low, intermediate and high risk procedures, having respectively <5%, 5–15% and >15% risk of PAF incidence) and patient characteristics [14,15,16,17,18]. If we consider lung surgery, the reported incidence ranges from 2% to 4% (wedge resection), to 10% to 15% (lobectomy), and to 20% to 30% (pneumonectomy) [19]. Data for esophageal cancer report a prevalence ranging between 4% and 10% [20,21]. 

**Figure 1:**
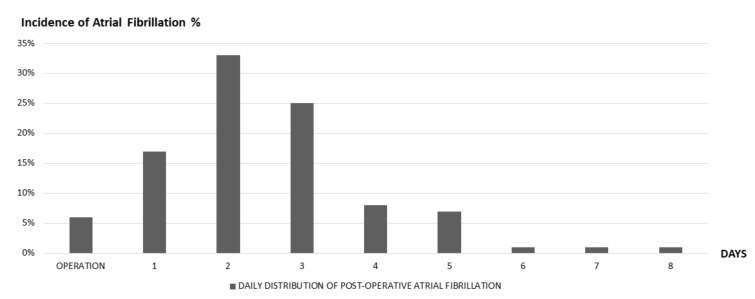
Daily distribution of post-operative atrial fibrillation—Pooled data with permission from [22,30,31], showing cumulative incidence of post-operative atrial fibrillation episodes, according to days after operation.

PAF usually peaks on post-operative days 2–4 (Figure 1), and as much as 98% of new-onset PAF episodes tend to resolve within 4 to 6 weeks [14,22]. 

PAF has multiple negative implications, including hemodynamic instability and acute HF, as well as embolic complications. 

According to Nojiri et al., PAF was the most frequent complication in the acute phase, and acute post-operative complications implicated a higher incidence of cardiovascular morbidity and mortality in the chronic phase after lung cancer surgery [23]. This is particularly true after lung resection, with higher rates of complications [24].

In the specific, PAF is associated with longer hospital stays (usually by a mean of 3 days), as well as increased morbidity (1.3–1.7% of incidence), mortality (up to 5.6–7.5%; Relative Risk 1.7–3.46) and consequent higher resource utilization [15,17,18,24,25,26,27,28]. Moreover, mortality after lung resection increases in patients with PAF [29,30,31].

However, the literature reports some contradictory findings [28]. In particular, considering the surgical risks, it remains unclear the exact contribution of the arrhythmia to mortality, especially if we account for other comorbidities often present in candidates to lung resection (including cardiovascular, renal, and respiratory conditions). 

Previous prospective findings from our group showed an increase in early AF occurrence after lung surgery; however, we did not report a negative influence on mortality or on relapses [28].

### 2.2. PAF and Thromboembolic Risk

The risk of thromboembolic events represents a major challenge in the field of thoracic oncology [32]. Patients with malignancies have a higher incidence of stroke, even without concurrent AF [33,34,35]. In particular, lung cancer confers an increased risk of ischemic stroke (Hazard Ratio (HR) 1.4, 95% Confidence Interval (CI) 1.3–1.5). Of note, a higher incidence of stroke was reported in patients with oncologic pulmonary disease; however, the ischemic stroke rate was higher within the first year of cancer diagnosis [33]. 

In summary, previous reports considering cancer patients with or without AF have demonstrated a higher rate of ischemic strokes and mortality linked to cerebro-vascular complications, also considering PAF cases [9,24]. 

### 2.3. Risk Factors, Triggers, and Pathophysiology of PAF

In the assessment of thoracic surgery risk, many pre-operative factors can be considered predictors for PAF. 

In an extensive retrospective analysis by Wu et al. [36], which reported an overall incidence of PAF of 3.27%, it was found that age, male gender, lung cancer, general anesthesia, open surgery, lobe resection, and duration of the operation resulted in risk factors of PAF. In another retrospective study by Muranishi et al., conducted on 593 patients [37], the overall incidence of PAF was 6.4%. In multivariate analysis, lymph node dissection (Odds Ratio (OR) Nodes-2/Nodes-0-1 = 3.06; 95% CI 1.06–10.9) was associated with PAF occurrence. Iwata et al. [38], in a recent comprehensive literature review and single-center case experience, found gender (male), resected lung size, high brain natriuretic peptide, and left ventricular increased filled pressures by echocardiography, as independent predictors for PAF. 

In a recent paper by Kavurmaci et al. [39], considering patients who underwent lung resection due to primary lung cancer, authors found that older age and chronic obstructive pulmonary disease (COPD) were significant predictors of PAF.

In this respect, thoracic surgical procedures may be divided into low (<5%), moderate (5–15%) and high (>15%) risk, according to their expected incidence of PAF, in order to enhance the preoperative risk stratification of patients (Table 1). Similarly, the presence of multiple factors (scoring system) implies an increased risk of PAF.

**Figure 2:**
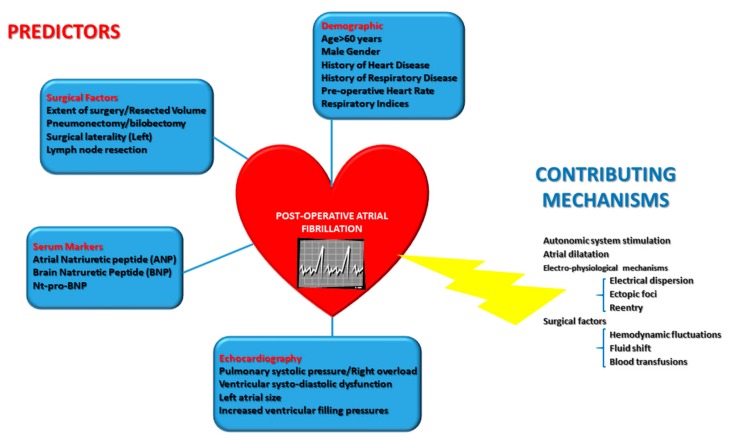
Graphical depiction of post-operative atrial fibrillation «milieu»—Independent predictors derived from principal studies from literature are in boxes, according to their type. Contributing mechanisms, including electro-physiological ones, are in blue.

So far, risk factors for PAF after cancer therapy are classified as acute (or triggers, i.e., direct surgical impact) and chronic factors (the result of a gradual process of remodeling) [40,41]. Generally speaking, most of the risk factors for PAF are similar to the non-surgical setting, including atrial fibrosis enhancing conditions (such as age), atrial enlargement, ischemia, pressure-volume overload, and heart failure [42,43,44]. They also include autonomic factors, such as increased catecholamine levels or vagal tone, in which fluctuations are commonly reported in the surgical setting [1,45] (Figure 2). More specifically, surgical procedures imply inflammation, a factor increasing the vulnerability of the atrial substrate to PAF [46].

In particular, higher catecholamine levels are reported in patients on pre-operative beta-blockers after a discontinuation of this therapy, respective to patients not receiving this class of drugs [42,47].

Diltiazem therapy has been found to reduce the rate of PAF [1,44,48,49].

From a pathophysiological standpoint, the mechanisms that initiate and sustain the arrhythmia comprise a vulnerable substrate and a trigger (such as pulmonary veins) to start AF [50]. In particular, pulmonary vein (PV) stumps in AF patients after a pneumonectomy represent sites of active electrical activity [51,52,53]. Previous reports identified a tight link between PAF development and activation of inflammatory cytokines [54,55], and cardiac infiltration of inflammatory cells is associated with PAF [56].

Furthermore, a strong relationship links post-operative hypoxia and PAF, and conditions predisposing to post-operative hypoxia might have a substantial role in PAF occurrence [57,58].

However, mechanical (i.e., surgical) conditions largely influence PAF triggering. In particular, the volume of pulmonary resection is a critical risk factor for the development of PAF [59]. In a recent analysis of patients who underwent surgery for lung cancer (stage I A), the vast majority of PAF was reported for lobectomy. Increasing age (HR 1.059, 95% CI 1.015–1.106, *p* < 0.01 for each age increase), surgical mode (lobectomy) (HR 5.734, 95% CI 1.350–24.361, *p* = 0.02) and Forced Expiratory Volume (FEV) 1.0% < 70% (HR 2.182, 95% CI 1.067–4.461, *p* = 0.03) were independent predictors of PAF at multivariate analysis [60].

Unlike cardiac surgery, there is no commonly implemented clinical risk score for the prediction of PAF after thoracic surgery [61].

In a retrospective study on 525 patients, preoperative CHA_2_DS_2_-VASc score could predict PAF in candidates to lobe resection. Age resulted the most relevant independent predictor, with patients with scores ≥5 having increased risk [62].

### 2.4. Implications of Chemotherapy

A relevant implication might be found in previous chemotherapic treatments. In fact, after chemotherapy, patients show a higher susceptibility to post-operative arrhythmias [63].

In a study on thoracic surgery (esophageal), Rice et al. [64] noted a 34% risk of atrial arrhythmias in subjects receiving previous chemotherapy. Additionally, a significant incidence of PAF was reported in surgical lung cases after induction therapy [65]. Moreover, in this context, inflammation may potentially explain this phenomenon [66].

### 2.5. Prediction of PAF

Prophylaxis against PAF in cardiovascular surgery has been highly emphasized, while in the field of lung cancer, surgery data remain limited. We mainly refer, according to randomized studies, to the use of beta-blockers, diltiazem, verapamil, amiodarone, and intravenous magnesium [14]. These strategies have reduced the incidence of PAF after lung resection with mixed results [14,29], but a substantial incidence of side effects has been reported. So far, recent guidelines highlight the limited evidence in recommending widespread prophylaxis for PAF and that the optimal preventive strategy should be tailored for each patient [14]. Moreover, an undiscriminating precautionary approach for all surgical cases may have an unfavorable risk/benefit profile (in particular, in terms of exposition to side effects). Identification of high-risk situations seems, so far, the best strategy. In this respect—despite the several studies conducted to identify independent predictors—the only widely validated risk factor, from a demographic point of view, is advanced age [67,68].

There are several potential biomarkers valuable for PAF risk prediction, and several of them, in particular, circulatory biomarkers, have been proposed. However, the existing data are conflicting: markers of collagen synthesis (pro-collagen family), extracellular matrix remodeling, inflammation, and profibrotic mediators (interleukins) appear to be promising in the stratification task. In particular, alterations in these markers, as reported in the literature, may reflect differences in the methodology, the populations included, the type of AF, the presence of left ventricular dysfunction, as well as demographic and therapeutic implications. Furthermore, the lack of specificity of markers of myocardial extracellular matrix remodeling might represent the most relevant issue [69].

### 2.6. The Potential Role of Natriuretic Peptides

At present, natriuretic peptides (NPs) are the most extensively and clinically validated biomarkers in the field of PAF. They are released from the myocardium in response to multiple physiological stimuli (among others, ischemic or inflammatory conditions, overload) [70,71], and numerous studies have demonstrated the independent predictive role of elevated pre-operative NP levels in terms of perioperative cardiovascular complications [72,73,74,75].

So far, the major international society guidelines for pre-operative cardiac risk assessment recommend a pre-operative NPs evaluation in high-risk surgery [76].

Recently, pooled data from a metanalysis showed that the addition of post-operative NP sampling enhanced risk stratification after non-cardiac surgery compared with a pre-operative sampling alone [77,78]. However, post-operative NPs fluctuations may often reflect intra-operative (drugs, hemodynamic, fluid shifts, etc.).

Cardinale et al. previously showed that increased perioperative Nt-pro-BNP levels predicted PAF in patients undergoing lung surgery [22]. Both preoperative and post-operative Nt-pro-BNP values were independent predictors of AF at multivariable analysis (relative risk, 27.9; 95% CI, 13.2–58.9; *p* < 0.001 for preoperative Nt-pro-BNP elevation; relative risk, 20.1; 95% CI, 5.8–69.4; *p* < 0.001 for post-operative Nt-pro-BNP elevation). These results were confirmed in a randomized trial by the same group, including 1116 patients. Patients showing a high Nt-pro-BNP value (29%) were enrolled. A prophylactic strategy with either metoprolol or losartan, in high-risk patients only (elevated Nt-pro-BNP concentrations), reduced the occurrence of PAF, with a low number needed-to-treat [79]. A lower incidence of PAF implied a shorter length of stay and less post-operative adverse events. So far, PAF was closely related to high Nt-pro-BNP concentrations, with a stronger association for pre-operative levels.

Many published data have further illustrated the role of increased levels of NPs as predictors of post-operative major adverse clinical events, specifically PAF, both in cardiac and non-cardiac surgery [67,73,77,80,81]. The patho-physiological link between high NPs levels and PAF occurrence remains unclear. NPs may increase in chronic conditions (among others, ageing) associated per se to PAF occurrence [80,82].

Elevated pre-operative NPs may be caused by right ventricular overload in patients with chronic respiratory diseases. A post-operative increase in Nt-pro-BNP may be related to an acute overload of the right ventricle after pulmonary resection, particularly if extensive. However, other mechanisms are possibly involved, since limited lung resections may also increase NP levels.

In our paper, even though both therapeutic strategies reduced natriuretic peptide concentrations in other clinical conditions rapidly [83,84], we did not observe a relevant decrease in Nt-pro-BNP [79], while Nt-pro-BNP levels increased after surgery. This suggests that the prevention of PAF is not purely mediated by a reduction in Nt-pro-BNP concentrations. Hence, increase in perioperative Nt-pro-BNP concentrations represent more of a risk marker than a risk factor of PAF. So far, a single cut-off value for NPs could be identified to select high-risk patients to candidate them to preventive therapy [85].

## 3. Acute Treatment

In general, the acute management of PAF should reflect recent clinical guidelines [1,14]. In particular, patients with unstable conditions should be emergently addressed to cardioversion. In the stable patient, either parenteral rate or rhythm control strategy can be employed; while in subjects with limited symptoms, an oral approach (rate control) can be used.

In a small study of candidate to lung resection, no difference was found between amiodarone and diltiazem therapy in terms of sinus rhythm recovery [86]. Still, nowadays, there are no specific studies to guide treatment for PAF after thoracic surgery, and the arrhythmia is often diagnosed and managed differently than cardiac surgery [87].

The most commonly used drug is amiodarone (generally delivered as an intravenous infusion) [88]. The use of intravenous flecainide in rapid bolus is often performed at our institution, with a good profile of safety and efficacy. However, when a rhythm-control strategy is necessary post-operatively, a tailored approach, with the intent to minimize anti-arrhythmic drug exposure, remains the most suitable approach.

### 3.1. Atrial Fibrillation Prophylaxis

Different drugs have been used for the prevention of PAF [89,90,91]. Several randomized controlled trials (RCTs) and meta-analyses have evaluated these substances, reporting a varying degree of success [29,42,92].

The role of medical prophylaxis remains controversial [68], and previous reports, including meta-analyses, often compared individual drugs with a placebo only. In a recent meta-analysis by Zhang et al. [93] considering 12 randomized trials, amiodarone was the most effective drug in preventing post-operative AF, while there were no significant prophylactic effects by magnesium, digoxin, or Calcium Channels Blockers (CCB). Patients under amiodarone therapy reported a lower incidence of PAF, and its use seemed to be safe with no significant complications.

In this respect, another recent network meta-analysis with trial sequential analysis, including 22 trials, showed a reduced incidence of PAF with medical prophylaxis (OR, 0.33; 95% CI 0.22–0.49). Unfortunately, short-term mortality was unchanged. From the trial sequential, sufficient evidence emerged in support of medical prophylaxis as a preventive strategy for PAF after thoracic surgery. In network meta-analysis, several drugs (including amiodarone, CCB, magnesium) reduced the risk of PAF compared with the placebo. Beta-blockers had the highest probability of being the most effective agents [94].

The use of beta-blockers in patients undergoing lung surgery, considering the presence of respiratory comorbidity as the hemodynamic implications, remains of concern [19]. So far, indiscriminate administration of such medications seems not to be justified. The majority of evidence stems from the POISE (Perioperative-Ischemic-Evaluation) trial, where long-acting formulation of metoprolol before surgery showed to reduce the incidence of PAF [95].

However, an increased incidence of stroke and mortality (likely due to post-operative hypotension) was reported, limiting this therapeutic approach in clinical routine [95].

Few studies have evaluated the efficacy of beta-blockers for post-operative prophylaxis of PAF, with promising findings [79,96,97].

ACEI and receptor blockers (ARBs) have many positive and mixed effects, including modification of sympathetic tone and inflammation, as a direct anti-arrhythmic property [98,99,100]. However, data on the efficacy of ACEI/ARBs for preventing PAF have been limited, particularly after lung resection [79,101].

Other potential preventive measures derived from the cardiothoracic surgery field include pre-operative statin use in statin naive patients [102,103,104,105], but results in this field are partially conflicting, particularly if we consider randomized trials not limited to lung surgery.

Less limited evidence is available for colchicine use outside cardiac surgery/AF ablation settings [106,107,108,109].

A recent study highlighted the role of olprinone, a specific phosphodiesterase III inhibitor, in PAF prevention [110]. In particular, continuous infusion of olprinone during lung surgery was safe and reduced the rate of PAF following lung resection in patients with increased pre-operative BNP concentrations.

No data in the field of lung surgery are available for fish oil supplements, which showed promising results in the field of PAF prevention in a cardiac surgery setting [111,112,113,114].

### 3.2. Anti-Thrombotic Profilaxys

A precise evaluation of the patient’s risk of stroke plays a central role in providing adequate anti-thrombotic protection, with a reasonable risk of bleeding. Gialdini et al. clearly showed a significant association of ischemic stroke and PAF, stronger for non-cardiac surgery operations [11].

For PAF episodes more extended than 48 h, the standard recommendations follow conventional guidelines, since the occurrence of stroke result increased. In these patients, the incidence of ischemic events is lower with appropriate anti-thrombotic management, despite an increased risk of bleeding [1,14]. However, the presence of multiple risk factors for stroke may influence the decision to treat a patient earlier than this conventional time range. The option of a brief anticoagulation period should be tailored for every single case, balancing the risk factor for bleeding and thromboembolism. Initiation of warfarin is usually safe in the first 24 h from surgery, unless an increased risk for bleeding is present.

Recently, an association between PAF and thrombo-embolic events at follow-up was found, comparable to non-valvular atrial fibrillation in terms of adverse events, which benefited from the long-term antithrombotic strategy introduced in the first 30 days after the onset of the episode [115].

In this context, as in a fast-track strategy to restore sinus rhythm with electrical cardioversion, heparin represents the most suitable option.

Anticoagulation is also recommended when cardioversion is the selected option. New oral anticoagulant drugs represent a current opportunity to manage atrial fibrillation, including dabigatran, edoxaban, rivaroxaban, and apixaban. When compared to conventional dicumarolic substances, these agents offer the advantage of not requiring monitoring of the INR, and resulted non-inferior in terms of stroke prevention, with an overall reduced bleeding rate. However, at present, their role in PAF has not been evaluated.

Although recent studies in patients with long term rhythm monitoring showed an association of brief atrial fibrillation episodes and ischemic stroke [116], it remains challenging to prescribe a long-term course of anticoagulation (>1 month) unless recurrent atrial fibrillation is clearly demonstrated or concomitant, multiple risk factors of stroke are present.

### 3.3. Long Term Implications of PAF

There is limited literature on post-discharge risks for thoracic patients, and data remain debated. In the field of cardiac surgery, the adverse impact of PAF in the follow-up has been demonstrated [117], and in patients undergoing esophagectomy, PAF represented an independent predictor of follow-up mortality at one year and long term [118].

However, previous studies showed how most cases of PAF result in self-limiting and, also, if diagnosed at discharge, usually have evidence of restored sinus rhythm by the time of the follow-up. Rena et al. [30] demonstrated that the vast majority of PAF resolved after hospital dismission, while in a study by Amar et al., 50% of episodes of PAF spontaneously converted to sinus rhythm in less than 24 h [119].

These data have been questioned more recently. In particular, recent papers showed how, in non-cardiac surgery, atrial fibrillation recurrences were 37.28% in patients with PAF and 1.51% in those without [11]. In recent work on 377 patients who underwent anatomical pulmonary resection, the persistence of one-month arrhythmia was 7% [120]. Even more recently, Higuchi et al. have shown a recurrence rate of 30% atrial fibrillation, mainly asymptomatic, in subjects undergoing cancer surgery, associated with the presence of arterial hypertension and renal failure [121].

Although the development of PAF resulted in a risk factor for mortality in cardiac surgery cases [122], the implications for patients after thoracic surgery remain, in part, mostly unknown.

There is no definite evidence to guide the duration of anti-arrhythmic PAF therapy after thoracic surgery. Since many AF episodes can occur asymptomatically, data on long term monitoring (Holter ECG), either on drug therapy or not, may be able to partially identify the real AF burden. A critical point is the intensity of ECG recording during follow-up [123].

Some ongoing clinical trials are evaluating the impact of prolonged perioperative rhythm monitoring in patients undergoing cancer surgery [124]. In the MONITOR-AF trial [125], in patients undergoing coronary artery bypass surgery monitoring with an implantable loop recorder after PAF showed a 60% recurrence rate of atrial fibrillation.

Data in the literature to guide anti-arrhythmic therapy are scarce, mostly derived from cardiac surgery settings [126,127].

In our experience (unpublished data) on prolonged, Holter-driven, amiodarone therapy, did not result superior to a limited therapeutic course.

## 4. Conclusions

Post-operative atrial fibrillation is of common diagnosis in the field of thoracic surgery, and it has a recognized epidemiological, as well as clinical, relevance. In particular, it represents the most recognizable and historical association between cancer and atrial arrhythmogenesis. Although several demographic, surgical, and clinical or imaging predictors may better stratify the risk for developing it, the studies conducted so far have often been retrospective, limited by a relatively low number of patients.

Pharmacological prophylaxis is a debated point, and substantial evidence is still lacking.

Similarly, specifical treatment strategies reflect those from current atrial fibrillation guidelines. If we consider the costs in terms of morbidity and mortality (including hemodynamic and thrombotic complications requiring a prolonged hospital stay), a tight and tailored approach for risk stratification, potentially including pre and post-operative assessment of biomarkers, is potentially useful. In particular, more extensive randomized studies should be conducted to develop stronger evidence in therapeutic and preventive strategies, as well as to define the real prognostic weight respective to other comorbidities and the long term implications.

## Figures and Tables

**Table 1:** Thoracic surgery and risk of developing post-operative atrial fibrillation.

Procedure Type/Surgical Risk	Low	Intermediate	High
**Minor Procedures**	Bronchoscopy +/− Biopsy		
	Tracheal Stenting		
	Thoracostomy Tube Placement		
	Pleurodesis		
**Moderate Procedures**	Tracheostomy	Simpaticectomy	
	Rigid Bronchoscopy		
	Mediastinoscopy		
	Toracoscopic Wedge Resection		
**Major Procedures**		Segmentectomy	Pleurectomy
			Lobectomy
			Transplant
			Fistula Repair
			Bullectomy
			Pneumonectomy
			Tracheal Resection
			Anterior Mediastinal Resection

Readapted with permission from [13].

## Abbreviations

ACEI	angiotensin-converting enzyme inhibitor
AF	atrial fibrillation
ARB	angiotensin receptor blocker
BNP	brain natriuretic peptide
CCB	calcium channel blockers
COPD	chronic obstructive pulmonary disease
ECG	electrocardiogram
FEV1	forced expiratory volume in the 1st second
HF	heart failure
LOE	level of evidence
NP	natriuretic peptide
Nt	N-terminal
PAF	post-operative atrial fibrillation
POISE	perioperative-ischemic-evaluation
PV	pulmonary veins
RCT	randomized controlled trials

## References

[1] Kirchhof P., Benussi S., Kotecha D., Ahlsson A., Atar D., Casadei B., Castella M., Diener H.C., Heidbuchel H., Hendriks J. (2016). 2016 ESC Guidelines for the management of atrial fibrillation developed in collaboration with EACTS. Eur. Heart J..

[2] Ferreira C., Providência R., Ferreira M.J., Gonçalves L.M. (2015). Atrial Fibrillation and Non-cardiovascular Diseases: A Systematic Review. Arq. Bras. Cardiol..

[3] Conen D., Wong J.A., Sandhu R.K., Cook N.R., Lee I.M., Buring J.E., Albert C.M. (2016). Risk of Malignant Cancer Among Women With New-Onset Atrial Fibrillation. JAMA Cardiol..

[4] Guzzetti S., Costantino G., Sada S., Fundarò C. (2002). Colorectal cancer and atrial fibrillation: A case-control study. Am. J. Med..

[5] Erichsen R., Christiansen C.F., Mehnert F., Weiss N.S., Baron J.A., Sørensen H.T. (2012). Colorectal cancer and risk of atrial fibrillation and flutter: A population-based case-control study. Intern. Emerg. Med..

[6] Hu Y.F., Liu C.J., Chang P.M.H., Tsao H.M., Lin Y.J., Chang S.L., Lo L.W., Tuan T.C., Li C.H., Chao T.F. (2013). Incident thromboembolism and heart failure associated with new-onset atrial fibrillation in cancer patients. Int. J. Cardiol..

[7] Cheng W.L., Kao Y.H., Chen S.A., Chen Y.J. (2016). Pathophysiology of cancer therapy-provoked atrial fibrillation. Int. J. Cardiol..

[8] Zamorano J.L., Lancellotti P., Rodriguez Muñoz D., Aboyans V., Asteggiano R., Galderisi M., Habib G., Lenihan D.J., Lip G.Y., Lyon A.R. (2016). 2016 ESC Position Paper on cancer treatments and cardiovascular toxicity developed under the auspices of the ESC Committee for Practice Guidelines: The Task Force for cancer treatments and cardiovascular toxicity of the European Society of Cardiology (ESC). Eur. Heart J..

[9] Farmakis D., Parissis J., Filippatos G. (2014). Insights into onco-cardiology: Atrial fibrillation in cancer. J. Am. Coll. Cardiol..

[10] Chelazzi C., Villa G., de Gaudio A.R. (2011). Postoperative atrial fibrillation. ISRN Cardiol..

[11] Gialdini G., Nearing K., Bhave P.D., Bonuccelli U., Iadecola C., Healey J.S., Kamel H. (2014). Perioperative atrial fibrillation and the long-term risk of ischemic stroke. JAMA.

[12] Dunning J., Treasure T., Versteegh M., Nashef S.A. (2006). Guidelines on the prevention and management of de novo atrial fibrillation after cardiac and thoracic surgery. Eur. J. Cardiothorac. Surg..

[13] Frendl G., Sodickson A.C., Chung M.K., Waldo A.L., Gersh B.J., Tisdale J.E., Calkins H., Aranki S., Kaneko T., Cassivi S. (2014). 2014 AATS guidelines for the prevention and management of perioperative atrial fibrillation and flutter for thoracic surgical procedures. Executive summary. J. Thorac. Cardiovasc. Surg..

[14] Frendl G., Sodickson A.C., Chung M.K., Waldo A.L., Gersh B.J., Tisdale J.E., Calkins H., Aranki S., Kaneko T., Cassivi S. (2014). 2014 AATS guidelines for the prevention and management of perioperative atrial fibrillation and flutter for thoracic surgical procedures. J. Thorac. Cardiovasc. Surg..

[15] Roselli E.E., Murthy S.C., Rice T.W., Houghtaling P.L., Pierce C.D., Karchmer D.P., Blackstone E.H. (2005). Atrial fibrillation complicating lung cancer resection. J. Thorac. Cardiovasc. Surg..

[16] Passman R.S., Gingold D.S., Amar D., Lloyd-Jones D., Bennett C.L., Zhang H., Rusch V.W. (2005). Prediction rule for atrial fibrillation after major noncardiac thoracic surgery. Ann. Thorac. Surg..

[17] Onaitis M., D’Amico T., Zhao Y., O’Brien S., Harpole D. (2010). Risk factors for atrial fibrillation after lung cancer surgery: Analysis of the Society of Thoracic Surgeons general thoracic surgery database. Ann. Thorac. Surg..

[18] Ivanovic J., Maziak D.E., Ramzan S., McGuire A.L., Villeneuve P.J., Gilbert S., Sundaresan R.S., Shamji F.M., Seely A.J. (2014). Incidence, severity and perioperative risk factors for atrial fibrillation following pulmonary resection. Interact. Cardiovasc. Thorac. Surg..

[19] Shrivastava V., Nyawo B., Dunning J., Morritt G. (2004). Is there a role for prophylaxis against atrial fibrillation for patients undergoing lung surgery?. Interact. Cardiovasc. Thorac. Surg..

[20] Siu C.W., Tung H.M., Chu K.W., Jim M.H., Lau C.P., Tse H.F. (2005). Prevalence and predictors of new-onset atrial fibrillation after elective surgery for colorectal cancer. Pacing Clin. Electrophysiol..

[21] Ojima T., Iwahashi M., Nakamori M., Nakamura M., Katsuda M., Iida T., Hayata K., Yamaue H. (2014). Atrial fibrillation after esophageal cancer surgery: An analysis of 207 consecutive patients. Surg. Today.

[22] Cardinale D., Colombo A., Sandri M.T., Lamantia G., Colombo N., Civelli M., Salvatici M., Veronesi G., Veglia F., Fiorentini C. (2007). Increased perioperative N-terminal pro-B-type natriuretic peptide levels predict atrial fibrillation after thoracic surgery for lung cancer. Circulation.

[23] Nojiri T., Inoue M., Takeuchi Y., Maeda H., Shintani Y., Sawabata N., Hamasaki T., Okumura M. (2015). Impact of cardiopulmonary complications of lung cancer surgery on long-term outcomes. Surg. Today.

[24] Imperatori A., Mariscalco G., Riganti G., Rotolo N., Conti V., Dominioni L. (2012). Atrial fibrillation after pulmonary lobectomy for lung cancer affects long-term survival in a prospective single-center study. J. Cardiothorac. Surg..

[25] Henri C., Giraldeau G., Dorais M., Cloutier A.S., Girard F., Noiseux N., Ferraro P., Rinfret S. (2012). Atrial fibrillation after pulmonary transplantation: Incidence, impact on mortality, treatment effectiveness, and risk factors. Circ. Arrhythm. Electrophysiol..

[26] Bhave P.D., Goldman L.E., Vittinghoff E., Maselli J., Auerbach A. (2012). Incidence, predictors, and outcomes associated with postoperative atrial fibrillation after major noncardiac surgery. Am. Heart J..

[27] Vaporciyan A.A., Correa A.M., Rice D.C., Roth J.A., Smythe W.R., Swisher S.G., Walsh G.L., Putnam J.B. (2004). Risk factors associated with atrial fibrillation after noncardiac thoracic surgery: Analysis of 2588 patients. J. Thorac. Cardiovasc. Surg..

[28] Cardinale D., Martinoni A., Cipolla C.M., Civelli M., Lamantia G., Fiorentini C., Mezzetti M. (1999). Atrial fibrillation after operation for lung cancer: Clinical and prognostic significance. Ann. Thorac. Surg..

[29] Riber L.P., Larsen T.B., Christensen T.D. (2014). Postoperative atrial fibrillation prophylaxis after lung surgery: Systematic review and meta-analysis. Ann. Thorac. Surg..

[30] Rena O., Papalia E., Oliaro A., Casadio C., Ruffini E., Filosso P., Sacerdote C., Maggi G. (2001). Supraventricular arrhythmias after resection surgery of the lung. Eur. J. Cardiothorac. Surg..

[31] Polanczyk C.A., Goldman L., Marcantonio E.R., Orav E.J., Lee T.H. (1998). Supraventricular arrhythmia in patients having noncardiac surgery: Clinical correlates and effect on length of stay. Ann. Intern. Med..

[32] Fitzpatrick T., Carrier M., le Gal G. (2017). Cancer, atrial fibrillation, and stroke. Thromb. Res..

[33] Chen P.C., Muo C.H., Lee Y.T., Yu Y.H., Sung F.C. (2011). Lung cancer and incidence of stroke: A population-based cohort study. Stroke.

[34] Cestari D.M., Weine D.M., Panageas K.S., Segal A.Z., DeAngelis L.M. (2004). Stroke in patients with cancer: Incidence and etiology. Neurology.

[35] Schwarzbach C.J., Schaefer A., Ebert A., Held V., Bolognese M., Kablau M., Hennerici M.G., Fatar M. (2012). Stroke and cancer: The importance of cancer-associated hypercoagulation as a possible stroke etiology. Stroke.

[36] Wu D.H., Xu M.Y., Mao T., Cao H., Wu D.J., Shen Y.F. (2012). Risk factors for intraoperative atrial fibrillation: A retrospective analysis of 10,563 lung operations in a single center. Ann. Thorac. Surg..

[37] Muranishi Y., Sonobe M., Menju T., Aoyama A., Chen-Yoshikawa T.F., Sato T., Date H. (2017). Atrial fibrillation after lung cancer surgery: Incidence, severity, and risk factors. Surg. Today.

[38] Iwata T., Nagato K., Nakajima T., Suzuki H., Yoshida S., Yoshino I. (2016). Risk factors predictive of atrial fibrillation after lung cancer surgery. Surg. Today.

[39] Kavurmaci O., Akcam T.I., Ergonul A.G., Turhan K., Cakan A., Cagirici U. (2018). Is the Risk of Postoperative Atrial Fibrillation Predictable in Patients Undergoing Surgery Due to Primary Lung Cancer?. Heart Lung. Circ..

[40] Finkel T., Serrano M., Blasco M.A. (2007). The common biology of cancer and ageing. Nature.

[41] Maesen B., Nijs J., Maessen J., Allessie M., Schotten U. (2012). Post-operative atrial fibrillation: A maze of mechanisms. Europace.

[42] Tisdale J.E., Wroblewski H.A., Kesler K.A. (2010). Prophylaxis of atrial fibrillation after noncardiac thoracic surgery. Semin. Thorac. Cardiovasc. Surg..

[43] Terzi A., Furlan G., Chiavacci P., Dal Corso B., Luzzani A., Dalla Volta S. (1996). Prevention of atrial tachyarrhythmias after non-cardiac thoracic surgery by infusion of magnesium sulfate. Thorac. Cardiovasc. Surg..

[44] Amar D., Roistacher N., Burt M., Reinsel R.A., Ginsberg R.J., Wilson R.S. (1995). Clinical and echocardiographic correlates of symptomatic tachydysrhythmias after noncardiac thoracic surgery. Chest.

[45] Schotten U., Verheule S., Kirchhof P., Goette A. (2011). Pathophysiological mechanisms of atrial fibrillation: A translational appraisal. Physiol. Rev..

[46] Dixit S. (2009). Atrial fibrillation after major thoracic surgery: New insights into underlying mechanisms. J. Am. Coll. Cardiol..

[47] Merritt R.E., Shrager J.B. (2012). Prophylaxis and management of atrial fibrillation after general thoracic surgery. Thorac. Surg. Clin..

[48] Amar D., Roistacher N., Burt M.E., Rusch V.W., Bains M.S., Leung D.H., Downey R.J., Ginsberg R.J. (1997). Effects of diltiazem versus digoxin on dysrhythmias and cardiac function after pneumonectomy. Ann. Thorac. Surg..

[49] Van Mieghem W., Tits G., Demuynck K., Lacquet L., Deneffe G., Tjandra-Maga T., Demedts M. (1996). Verapamil as prophylactic treatment for atrial fibrillation after lung operations. Ann. Thorac. Surg..

[50] Akoum N., Daccarett M., McGann C., Segerson N., Vergara G., Kuppahally S., Badger T., Burgon N., Haslam T., Kholmovski E. (2011). Atrial fibrosis helps select the appropriate patient and strategy in catheter ablation of atrial fibrillation: A DE-MRI guided approach. J. Cardiovasc. Electrophysiol..

[51] Kanmanthareddy A., Vallakati A., Reddy Yeruva M.A.D.H.U., Dixit S., Di Biase L., Mansour M., Boolani H., Gunda S., Bunch T.J., Day J.D. (2015). Pulmonary vein isolation for atrial fibrillation in the postpneumonectomy population: A feasibility, safety, and outcomes study. J. Cardiovasc. Electrophysiol..

[52] Haissaguerre M., Jaïs P., Shah D.C., Takahashi A., Hocini M., Quiniou G., Garrigue S., Le Mouroux A., Le Métayer P., Clémenty J. (1998). Spontaneous initiation of atrial fibrillation by ectopic beats originating in the pulmonary veins. N. Engl. J. Med..

[53] Jais P., Hocini M., Macle L., Choi K.J., Deisenhofer I., Weerasooriya R., Shah D.C., Garrigue S., Raybaud F., Scavee C. (2002). Distinctive electrophysiological properties of pulmonary veins in patients with atrial fibrillation. Circulation.

[54] Chung M.K., Martin D.O., Sprecher D., Wazni O., Kanderian A., Carnes C.A., Bauer J.A., Tchou P.J., Niebauer M.J., Natale A. (2001). C-reactive protein elevation in patients with atrial arrhythmias: Inflammatory mechanisms and persistence of atrial fibrillation. Circulation.

[55] Aviles R.J., Martin D.O., Apperson-Hansen C., Houghtaling P.L., Rautaharju P., Kronmal R.A., Tracy R.P., Van Wagoner D.R., Psaty B.M., Lauer M.S. (2003). Inflammation as a risk factor for atrial fibrillation. Circulation.

[56] Hu Y.-F., Chen Y.J., Lin Y.J., Chen S.A. (2015). Inflammation and the pathogenesis of atrial fibrillation. Nat. Rev. Cardiol..

[57] Lohani K.R., Nandipati K.C., Rollins S.E., Fetten K., Lee T.H., Pallati P.K., Yamamoto S.R., Mittal S.K. (2015). Transthoracic approach is associated with increased incidence of atrial fibrillation after esophageal resection. Surg. Endosc..

[58] Ma J.Y., Wang Y., Zhao Y.F., Wu Z., Liu L.X., Kou Y.L., Yang J.J. (2006). Atrial fibrillation after surgery for esophageal carcinoma: Clinical and prognostic significance. World J. Gastroenterol..

[59] De Decker K., Jorens P.G., van Schil P. (2003). Cardiac complications after noncardiac thoracic surgery: An evidence-based current review. Ann. Thorac. Surg..

[60] Ueda T., Suzuki K., Matsunaga T., Takamochi K., Oh S. (2018). Postoperative atrial fibrillation is less frequent in pulmonary segmentectomy compared with lobectomy. Gen. Thorac. Cardiovasc. Surg..

[61] Mariscalco G., Biancari F., Zanobini M., Cottini M., Piffaretti G., Saccocci M., Banach M., Beghi C., Angelini G.D. (2014). Bedside tool for predicting the risk of postoperative atrial fibrillation after cardiac surgery: The POAF score. J. Am. Heart Assoc..

[62] Lee C.T., Strauss D.M., Stone L.E., Stoltzfus J.C., Puc M.M., Burfeind W.R. (2019). Preoperative CHA2DS2-VASc Score Predicts Postoperative Atrial Fibrillation after Lobectomy. Thorac. Cardiovasc. Surg..

[63] Guglin M., Aljayeh M., Saiyad S., Ali R., Curtis A.B. (2009). Introducing a new entity: Chemotherapy-induced arrhythmia. Europace.

[64] Rice D.C., Correa A.M., Vaporciyan A.A., Sodhi N., Smythe W.R., Swisher S.G., Walsh G.L., Putnam J.B., Komaki R., Ajani J.A. (2005). Preoperative chemoradiotherapy prior to esophagectomy in elderly patients is not associated with increased morbidity. Ann. Thorac. Surg..

[65] Peer M., Stav D., Cyjon A., Sandbank J., Vasserman M., Haitov Z., Sasson L., Schreiber L., Ezri T., Priel I.E. (2015). Morbidity and mortality after major pulmonary resections in patients with locally advanced stage IIIA non-small cell lung carcinoma who underwent induction therapy. Heart Lung Circ..

[66] Tsujimoto H., Ono S., Chochi K., Sugasawa H., Ichikura T., Mochizuki H. (2006). Preoperative chemoradiotherapy for esophageal cancer enhances the postoperative systemic inflammatory response. Jpn. J. Clin. Oncol..

[67] Cai G.L., Chen J., Hu C.B., Yan M.L., Xu Q.H., Yan J. (2014). Value of plasma brain natriuretic peptide levels for predicting postoperative atrial fibrillation: A systemic review and meta-analysis. World J. Surg..

[68] Amar D. (2016). Postoperative atrial fibrillation: Is there a need for prevention?. J. Thorac. Cardiovasc. Surg..

[69] Turagam M.K., Mirza M., Werner P.H., Sra J., Kress D.C., Tajik A.J., Jahangir A. (2016). Circulating Biomarkers Predictive of Postoperative Atrial Fibrillation. Cardiol. Rev..

[70] Struthers A., Lang C. (2007). The potential to improve primary prevention in the future by using BNP/N-BNP as an indicator of silent ‘pancardiac’ target organ damage: BNP/N-BNP could become for the heart what microalbuminuria is for the kidney. Eur. Heart J..

[71] Clerico A., Giannoni A., Vittorini S., Passino C. (2011). Thirty years of the heart as an endocrine organ: Physiological role and clinical utility of cardiac natriuretic hormones. Am. J. Physiol. Heart Circ. Physiol..

[72] Rodseth R.N., Buse G.A.L., Bolliger D., Burkhart C.S., Cuthbertson B.H., Gibson S.C., Mahla E., Leibowitz D.W., Biccard B.M. (2011). The predictive ability of pre-operative B-type natriuretic peptide in vascular patients for major adverse cardiac events: An individual patient data meta-analysis. J. Am. Coll. Cardiol..

[73] Karthikeyan G., Moncur R.A., Levine O., Heels-Ansdell D., Chan M.T., Alonso-Coello P., Yusuf S., Sessler D., Villar J.C., Berwanger O. (2009). Is a pre-operative brain natriuretic peptide or N-terminal pro-B-type natriuretic peptide measurement an independent predictor of adverse cardiovascular outcomes within 30 days of noncardiac surgery? A systematic review and meta-analysis of observational studies. J. Am. Coll. Cardiol..

[74] Cardinale D., Cosentino N., Moltrasio M., Sandri M.T., Petrella F., Colombo A., Bacchiani G., Tessitore A., Bonomi A., Veglia F. (2018). Acute kidney injury after lung cancer surgery: Incidence and clinical relevance, predictors, and role of N-terminal pro B-type natriuretic peptide. Lung Cancer.

[75] Biccard B.M., Lurati Buse G.A., Burkhart C., Cuthbertson B.H., Filipovic M., Gibson S.C., Mahla E., Leibowitz D.W., Rodseth R.N. (2012). The influence of clinical risk factors on pre-operative B-type natriuretic peptide risk stratification of vascular surgical patients. Anaesthesia.

[76] Poldermans D., Hoeks S.E., Feringa H.H. (2008). Pre-operative risk assessment and risk reduction before surgery. J. Am. Coll. Cardiol..

[77] Rodseth R.N., Biccard B.M., Le Manach Y., Sessler D.I., Buse G.A.L., Thabane L., Schutt R.C., Bolliger D., Cagini L., Cardinale D. (2014). The prognostic value of pre-operative and post-operative B-type natriuretic peptides in patients undergoing noncardiac surgery: B-type natriuretic peptide and N-terminal fragment of pro-B-type natriuretic peptide: A systematic review and individual patient data meta-analysis. J. Am. Coll. Cardiol..

[78] Rodseth R.N., Biccard B.M., Chu R., Buse G.A.L., Thabane L., Bakhai A., Bolliger D., Cagini L., Cahill T.J., Cardinale D. (2013). Postoperative B-type natriuretic peptide for prediction of major cardiac events in patients undergoing noncardiac surgery: Systematic review and individual patient meta-analysis. Anesthesiology.

[79] Cardinale D., Sandri M.T., Colombo A., Salvatici M., Tedeschi I., Bacchiani G., Beggiato M., Meroni C.A., Civelli M., Lamantia G. (2016). Prevention of Atrial Fibrillation in High-risk Patients Undergoing Lung Cancer Surgery: The PRESAGE Trial. Ann. Surg..

[80] Amar D., Zhang H., Shi W., Downey R.J., Bains M.S., Park B.J., Flores R., Rizk N., Thaler H.T., Rusch V.W. (2012). Brain natriuretic peptide and risk of atrial fibrillation after thoracic surgery. J. Thorac. Cardiovasc. Surg..

[81] Wazni O.M., Martin D.O., Marrouche N.F., Latif A.A., Ziada K., Shaaraoui M., Almahameed S., Schweikert R.A., Saliba W.I., Gillinov A.M. (2004). Plasma B-type natriuretic peptide levels predict postoperative atrial fibrillation in patients undergoing cardiac surgery. Circulation.

[82] Nojiri T., Maeda H., Takeuchi Y., Funakoshi Y., Kimura T., Maekura R., Yamamoto K., Okumura M. (2010). Predictive value of B-type natriuretic peptide for postoperative atrial fibrillation following pulmonary resection for lung cancer. Eur. J. Cardiothorac. Surg..

[83] Yoshimura M., Mizuno Y., Nakayama M., Sakamoto T., Sugiyama S., Kawano H., Soejima H., Hirai N., Saito Y., Nakao K. (2002). B-type natriuretic peptide as a marker of the effects of enalapril in patients with heart failure. Am. J. Med..

[84] Johnson W., Omland T., Hall C., Lucas C., Myking O.L., Collins C., Pfeffer M., Rouleau J.L., Stevenson L.W. (2002). Neurohormonal activation rapidly decreases after intravenous therapy with diuretics and vasodilators for class IV heart failure. J. Am. Coll. Cardiol..

[85] Salvatici M., Cardinale D., Spaggiari L., Veglia F., Tedesco C.C., Solli P., Cipolla C.M., Zorzino L., Passerini R., Riggio D. (2010). Atrial fibrillation after thoracic surgery for lung cancer: Use of a single cut-off value of N-terminal pro-B type natriuretic peptide to identify patients at risk. Biomarkers.

[86] Bobbio A., Caporale D., Internullo E., Ampollini L., Bettati S., Rossini E., Carbognani P., Rusca M. (2007). Postoperative outcome of patients undergoing lung resection presenting with new-onset atrial fibrillation managed by amiodarone or diltiazem. Eur. J. Cardiothorac. Surg..

[87] Danelich I.M., Lose J.M., Wright S.S., Asirvatham S.J., Ballinger B.A., Larson D.W., Lovely J.K. (2014). Practical management of postoperative atrial fibrillation after noncardiac surgery. J. Am. Coll. Surg..

[88] Boriani G., Ferruzzi L., Corti B., Ruffato A., Gavelli G., Mattioli S. (2012). Short-term onset of fatal pulmonary toxicity in a patient treated with intravenous amiodarone for post-operative atrial fibrillation. Int. J. Cardiol..

[89] Riber L.P., Christensen T.D., Jensen H.K., Hoejsgaard A., Pilegaard H.K. (2012). Amiodarone significantly decreases atrial fibrillation in patients undergoing surgery for lung cancer. Ann. Thorac. Surg..

[90] Nojiri T., Yamamoto K., Maeda H., Takeuchi Y., Funakoshi Y., Inoue M., Okumura M. (2012). Effect of low-dose human atrial natriuretic peptide on postoperative atrial fibrillation in patients undergoing pulmonary resection for lung cancer: A double-blind, placebo-controlled study. J. Thorac. Cardiovasc. Surg..

[91] Ciszewski P., Tyczka J., Nadolski J., Roszak M., Dyszkiewicz W. (2013). Comparative efficacy and usefulness of acebutolol and diltiazem for the prevention of atrial fibrillation during perioperative time in patients undergoing pulmonary resection. Thorac. Cardiovasc. Surg..

[92] Sedrakyan A., Treasure T., Browne J., Krumholz H., Sharpin C., van der Meulen J. (2005). Pharmacologic prophylaxis for postoperative atrial tachyarrhythmia in general thoracic surgery: Evidence from randomized clinical trials. J. Thorac. Cardiovasc. Surg..

[93] Zhang L., Gao S. (2016). Systematic Review and Meta-analysis of Atrial Fibrillation Prophylaxis After Lung Surgery. J. Cardiovasc. Pharmacol..

[94] Zhao B.C., Huang T.Y., Deng Q.W., Liu W.F., Liu J., Deng W.T., Liu K.X., Li C. (2017). Prophylaxis Against Atrial Fibrillation After General Thoracic Surgery: Trial Sequential Analysis and Network Meta-Analysis. Chest.

[95] POISE Study Group (2008). Effects of extended-release metoprolol succinate in patients undergoing non-cardiac surgery (POISE trial): A randomised controlled trial. Lancet.

[96] Jakobsen C.J., Bille S., Ahlburg P., Rybro L., Hjortholm K., Andresen E.B. (1997). Perioperative metoprolol reduces the frequency of atrial fibrillation after thoracotomy for lung resection. J. Cardiothorac. Vasc. Anesth..

[97] Bayliff C.D., Massel D.R., Inculet R.I., Malthaner R.A., Quinton S.D., Powell F.S., Kennedy R.S. (1999). Propranolol for the prevention of postoperative arrhythmias in general thoracic surgery. Ann. Thorac. Surg..

[98] Reinhart K., Baker W.L., Siv M.L. (2011). Beyond the guidelines: New and novel agents for the prevention of atrial fibrillation after cardiothoracic surgery. J. Cardiovasc. Pharmacol. Ther..

[99] Jibrini M.B., Molnar J., Arora R.R. (2008). Prevention of atrial fibrillation by way of abrogation of the renin-angiotensin system: A systematic review and meta-analysis. Am. J. Ther..

[100] Ozaydin M., Dede O., Varol E., Kapan S., Turker Y., Peker O., Duver H., Ibrisim E. (2008). Effect of renin-angiotensin aldosteron system blockers on postoperative atrial fibrillation. Int. J. Cardiol..

[101] DiNicolantonio J.J., Beavers C.J., Menezes A.R., Lavie C.J., O’Keefe J.H., Meier P., Vorobcsuk A., Aradi D., Komócsi A., Chatterjee S. (2014). Meta-analysis comparing carvedilol versus metoprolol for the prevention of postoperative atrial fibrillation following coronary artery bypass grafting. Am. J. Cardiol..

[102] Amar D., Zhang H., Heerdt P.M., Park B., Fleisher M., Thaler H.T. (2005). Statin use is associated with a reduction in atrial fibrillation after noncardiac thoracic surgery independent of C-reactive protein. Chest.

[103] Blanchard L., Collard C.D. (2007). Non-antiarrhythmic agents for prevention of postoperative atrial fibrillation: Role of statins. Curr. Opin. Anaesthesiol..

[104] De Waal B.A., Buise M.P., van Zundert A.A. (2015). Perioperative statin therapy in patients at high risk for cardiovascular morbidity undergoing surgery: A review. Br. J. Anaesth..

[105] Bang C.N., Greve A.M., Abdulla J., Køber L., Gislason G.H., Wachtell K. (2013). The preventive effect of statin therapy on new-onset and recurrent atrial fibrillation in patients not undergoing invasive cardiac interventions: A systematic review and meta-analysis. Int. J. Cardiol..

[106] Imazio M., Belli R., Brucato A., Ferrazzi P., Patrini D., Martinelli L., Polizzi V., Cemin R., Leggieri A., Caforio A.L. (2013). Rationale and design of the COlchicine for Prevention of the Post-pericardiotomy Syndrome and Post-operative Atrial Fibrillation (COPPS-2 trial): A randomized, placebo-controlled, multicenter study on the use of colchicine for the primary prevention of the postpericardiotomy syndrome, postoperative effusions, and postoperative atrial fibrillation. Am. Heart J..

[107] Imazio M., Brucato A., Ferrazzi P., Pullara A., Adler Y., Barosi A., Caforio A.L., Cemin R., Chirillo F., Comoglio C. (2014). Colchicine for prevention of postpericardiotomy syndrome and postoperative atrial fibrillation: The COPPS-2 randomized clinical trial. JAMA.

[108] Imazio M., Brucato A., Ferrazzi P., Rovere M.E., Gandino A., Cemin R., Ferrua S., Belli R., Maestroni S., Simon C. (2011). Colchicine reduces postoperative atrial fibrillation: Results of the Colchicine for the Prevention of the Postpericardiotomy Syndrome (COPPS) atrial fibrillation substudy. Circulation.

[109] Worden J.C., Asare K. (2014). Postoperative atrial fibrillation: Role of inflammatory biomarkers and use of colchicine for its prevention. Pharmacotherapy.

[110] Nojiri T., Yamamoto K., Maeda H., Takeuchi Y., Ose N., Susaki Y., Inoue M., Okumura M. (2015). A Double-Blind Placebo-Controlled Study of the Effects of Olprinone, a Specific Phosphodiesterase III Inhibitor, for Preventing Postoperative Atrial Fibrillation in Patients Undergoing Pulmonary Resection for Lung Cancer. Chest.

[111] Masson S., Wu J.H., Simon C., Barlera S., Marchioli R., Mariani J., Macchia A., Lombardi F., Vago T., Aleksova A. (2015). Circulating cardiac biomarkers and postoperative atrial fibrillation in the OPERA trial. Eur. J. Clin. Invest..

[112] Mozaffarian D., Marchioli R., Macchia A., Silletta M.G., Ferrazzi P., Gardner T.J., Latini R., Libby P., Lombardi F., O’Gara P.T. (2012). Fish oil and postoperative atrial fibrillation: The Omega-3 Fatty Acids for Prevention of Post-operative Atrial Fibrillation (OPERA) randomized trial. JAMA.

[113] Mozaffarian D., Marchioli R., Macchia A., Silletta M.G., Ferrazzi P., Gardner T.J., Latini R., Libby P., Lombardi F., O’Gara P.T. (2013). Fish oil and post-operative atrial fibrillation: A meta-analysis of randomized controlled trials. J. Am. Coll. Cardiol..

[114] Wu J.H., Marchioli R., Silletta M.G., Masson S., Sellke F.W., Libby P., Milne G.L., Brown N.J., Lombardi F., Damiano R.J. (2015). Oxidative Stress Biomarkers and Incidence of Postoperative Atrial Fibrillation in the Omega-3 Fatty Acids for Prevention of Postoperative Atrial Fibrillation (OPERA) Trial. J. Am. Heart Assoc..

[115] Butt J.H., Olesen J.B., Havers-Borgersen E., Gundlund A., Andersson C., Gislason G.H., Torp-Pedersen C., Køber L., Fosbøl E.L. (2018). Risk of Thromboembolism Associated With Atrial Fibrillation Following Noncardiac Surgery. J. Am. Coll. Cardiol..

[116] Boriani G., Glotzer T.V., Santini M., West T.M., De Melis M., Sepsi M., Gasparini M., Lewalter T., Camm J.A., Singer D.E. (2014). Device-detected atrial fibrillation and risk for stroke: An analysis of >10,000 patients from the SOS AF project (Stroke preventiOn Strategies based on Atrial Fibrillation information from implanted devices). Eur. Heart J..

[117] Verma A., Bhatt D.L., Verma S. (2018). Long-Term Outcomes of Post-Operative Atrial Fibrillation: Guilty as Charged. J. Am. Coll. Cardiol..

[118] Chin J.H., Moon Y.J., Jo J.Y., Han Y.A., Kim H.R., Lee E.H., Choi I.C. (2016). Association between Postoperatively Developed Atrial Fibrillation and Long-Term Mortality after Esophagectomy in Esophageal Cancer Patients: An Observational Study. PLoS ONE.

[119] Amar D. (2002). Postoperative atrial fibrillation. Heart Dis..

[120] Garner M., Routledge T., King J.E., Pilling J.E., Veres L., Harrison-Phipps K., Bille A., Harling L. (2017). New-onset atrial fibrillation after anatomic lung resection: Predictive factors, treatment and follow-up in a UK thoracic centre. Interact. Cardiovasc. Thorac. Surg..

[121] Higuchi S., Kabeya Y., Matsushita K., Arai N., Tachibana K., Tanaka R., Kawachi R., Takei H., Suzuki Y., Kogure M. (2019). Perioperative Atrial Fibrillation in Noncardiac Surgeries for Malignancies and One-Year Recurrence. Can. J. Cardiol..

[122] Ahlsson A., Fengsrud E., Bodin L., Englund A. (2010). Postoperative atrial fibrillation in patients undergoing aortocoronary bypass surgery carries an eightfold risk of future atrial fibrillation and a doubled cardiovascular mortality. Eur. J. Cardiothorac. Surg..

[123] Lowres N., Mulcahy G., Gallagher R., Ben Freedman S., Marshman D., Kirkness A., Orchard J., Neubeck L. (2016). Self-monitoring for atrial fibrillation recurrence in the discharge period post-cardiac surgery using an iPhone electrocardiogram. Eur. J. Cardiothorac. Surg..

[124] Higuchi S., Kabeya Y., Matsushita K., Tachibana K., Kawachi R., Takei H., Suzuki Y., Abe N., Imanishi Y., Moriyama K. (2018). The study protocol for PREDICT AF RECURRENCE: A PRospEctive cohort stuDy of surveIllanCe for perioperaTive Atrial Fibrillation RECURRENCE in major non-cardiac surgery for malignancy. BMC Cardiovasc. Disord..

[125] El-Chami M.F., Merchant F.M., Smith P., Levy M., Nelms A.G., Merlino J., Puskas J., Leon A.R. (2016). Management of New-Onset Postoperative Atrial Fibrillation Utilizing Insertable Cardiac Monitor Technology to Observe Recurrence of AF (MONITOR-AF). Pacing Clin. Electrophysiol..

[126] Landymore R.W., Howell F. (1991). Recurrent atrial arrhythmias following treatment for postoperative atrial fibrillation after coronary bypass operations. Eur. J. Cardiothorac. Surg..

[127] Yilmaz A.T., Demírkiliç U., Arslan M., Kurulay E., Özal E., Tatar H., Öztürk Ö.Y. (1996). Long-term prevention of atrial fibrillation after coronary artery bypass surgery: Comparison of quinidine, verapamil, and amiodarone in maintaining sinus rhythm. J. Card. Surg..

